# The effect of well-characterized, very low-dose x-ray radiation on fibroblasts

**DOI:** 10.1371/journal.pone.0190330

**Published:** 2018-01-04

**Authors:** Katelyn Truong, Suzanne Bradley, Bryana Baginski, Joseph R. Wilson, Donald Medlin, Leon Zheng, R. Kevin Wilson, Matthew Rusin, Endre Takacs, Delphine Dean

**Affiliations:** 1 Department of Bioengineering, Clemson University, Clemson, South Carolina, United States of America; 2 Department of Physics and Astronomy, Clemson University, Clemson, South Carolina, United States of America; Northwestern University Feinberg School of Medicine, UNITED STATES

## Abstract

The purpose of this study is to determine the effects of low-dose radiation on fibroblast cells irradiated by spectrally and dosimetrically well-characterized soft x-rays. To achieve this, a new cell culture x-ray irradiation system was designed. This system generates characteristic fluorescent x-rays to irradiate the cell culture with x-rays of well-defined energies and doses. 3T3 fibroblast cells were cultured in cups with Mylar^®^ surfaces and were irradiated for one hour with characteristic iron (Fe) K x-ray radiation at a dose rate of approximately 550 μGy/hr. Cell proliferation, total protein analysis, flow cytometry, and cell staining were performed on fibroblast cells to determine the various effects caused by the radiation. Irradiated cells demonstrated increased proliferation and protein production compared to control samples. Flow cytometry revealed that a higher percentage of irradiated cells were in the G_0_/G_1_ phase of the cell cycle compared to control counterparts, which is consistent with other low-dose studies. Cell staining results suggest that irradiated cells maintained normal cell functions after radiation exposure, as there were no qualitative differences between the images of the control and irradiated samples. The result of this study suggest that low-dose soft x-ray radiation might cause an initial pause, followed by a significant increase, in proliferation. An initial “pause” in cell proliferation could be a protective mechanism of the cells to minimize DNA damage caused by radiation exposure. The new cell irradiation system developed here allows for unprecedented control over the properties of the x-rays given to the cell cultures. This will allow for further studies on various cell types with known spectral distribution and carefully measured doses of radiation, which may help to elucidate the mechanisms behind varied cell responses to low-dose x-rays reported in the literature.

## Introduction

Ionizing x-ray radiation exposure can cause DNA damage and the development of cancer, yet people are constantly exposed to x-rays and other forms of radiation from many different sources [1]. These sources include naturally occurring background radiation, cosmic radiation during space travel, diagnostic medical imaging such as x-rays and CT scans, radiation therapy for cancer treatment, and even from disaster areas like Fukushima [1–7]. Since the 1980’s, medical imaging has become an integral part of healthcare diagnostics, exposing patients to radiation at ever-increasing frequencies [3, 8]. Recent *in vitro* experiments support the hypothesis that the radiation environment of space could also contribute to the long-term physiological changes astronauts experience after missions [9, 10]. Because radiation exposure is so ubiquitous and can vary greatly across populations, it is important to fully understand the effects of low and high dose radiation on all human tissue and cell types to recognize and prevent detrimental effects.

Exposure from different sources has various total doses, exposure rates, linear energy transfer, and spectral features, which make certain aspects more harmful or more beneficial than others [11]. Medical radiation sources such as linear accelerators used for cancer treatment are designed to destroy cancerous tissue by the use of focused high doses [several 10s of Gy over the course of a treatment) of high-energy radiation (in the MeV photon range) while sparing healthy tissue in the regions of low dose [12]. Diagnostic x-ray sources operate with lower-energy (around 100 keV) radiation which has higher linear energy transfer (LET) than therapeutic devices, but these x-ray sources are considered to have acceptable risk of damage due to low-dose (on the order of 0.1 mGy to 400 mGy) employed [2, 8]. In order to minimize the unwanted damage that ionizing radiation sources produce, the physical and biological processes involved need to be understood with properly characterized systematic measurements, especially in the low-dose region [4, 13, 14].

Research on the effect of low-dose radiation on cells has shown wide ranges of results due to the variation in cell types, radiation source, and doses [14–18]. Some studies have shown no effect of low-dose (<0.1 Gy) radiation on cells [19, 20], but others have suggested that low-dose x-ray radiation has positive effects on the proliferation of cell types such as fibroblasts and osteoblasts, as well as in animal models [16, 18, 21].

Our study aimed to determine the effect of low dose (here approximately 550 μGy) x-ray radiation on fibroblast cells *in vitro* using characteristic fluorescent x-rays with well-defined energies and doses. Well-defined characteristic x-rays produced by a novel x-ray fluorescence irradiation device were utilized to aid the physical characterization of the radiation, as standard x-ray tube sources produce a mix of Bremsstrahlung and characteristic emissions [22]. Characteristic x-rays have a narrow wavelength band; therefore, the type and dose of x-rays given to the cells in this study are more controlled than previous studies using standard x-ray tubes or electron beam based sources [16, 18, 23–26].

Fibroblasts were chosen for this study due to their presence in connective tissues and critical role in secreting wound-healing proteins in the presence of tissue damage [27]. *In vitro*, the NIH 3T3 fibroblast cell line is well studied and experiences proliferation at a very high rate, which facilitates observation of the effects of irradiation on proliferation and serves as an ideal subject for testing the well characterized x-rays produced by the novel irradiation device in this study. Since low-dose radiation is known to affect the cell cycle of some cell types [16], we hypothesized that the exposure will accelerate the fibroblast cell cycle leading to increased proliferation of cells over time.

## Materials and methods

### Cell culture and Mylar^®^ cup assembly

**Fig 1:**
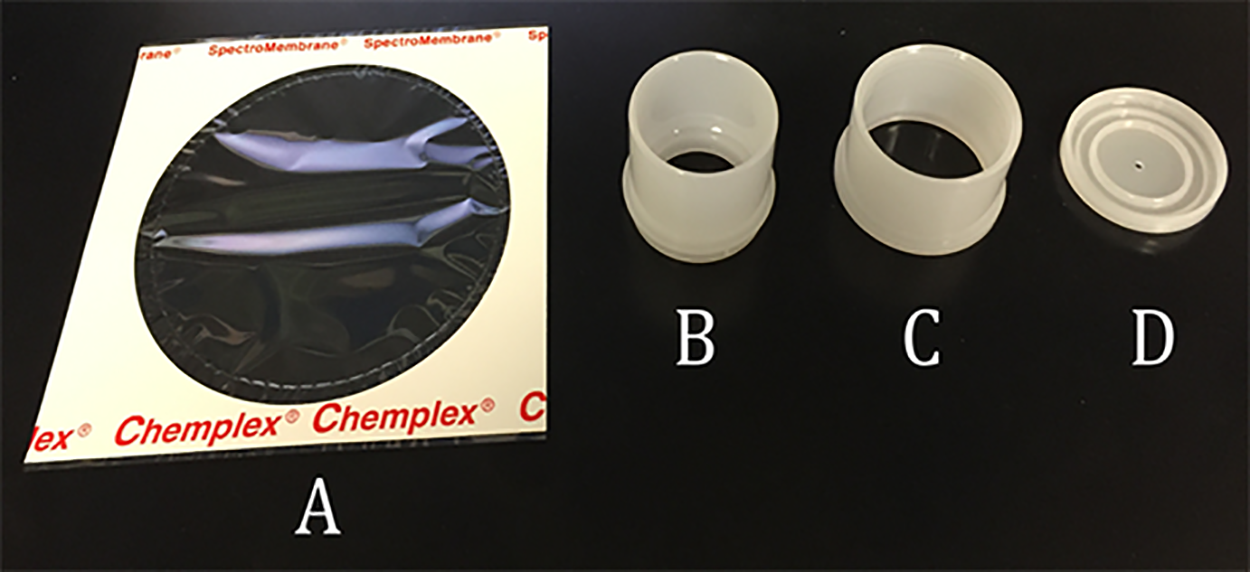
Mylar^®^ and individual cup components that make up the cell culture system. Mylar^®^ (A) provided a surface that allowed cells to grow in the same manner as if grown on standard tissue culture plastic, while also being thin enough to allow x-rays to pass through without significant attenuation. The Mylar^®^ was pressed between the inner (B) and outer (C) cup components with a ventilation cap (D) to allow proper air-flow.

NIH 3T3 mouse fibroblast cells were cultured on a 6 μm thick Mylar^®^ sheet in a self-assembled high-density polyethylene (HDPE) cup (Chemplex Industries Inc., Palm City, FL; Fig 1). Mylar^®^ was chosen as the surface material because it allowed for minimal attenuation of the x-ray beam during irradiation. This arrangement is a critical component of our study: x-ray absorption in plating substances changes with material composition and thickness, and can induce a cascade of secondary particles reaching the cell culture samples. The precise characterization of the radiation received by the cell cultures is one of the key elements for the systematic understanding of radiation effects.

The cells were plated at a density of approximately 1,000 cells/cm^2^ with 2 mL of standard cell culture media containing high-glucose Dulbecco’s Modified Eagle’s Medium (DMEM), 10% Fetal Bovine Serum (FBS), and 1% penicillin-streptomycin. Cells were incubated in standard culture conditions (5% CO_2_, 37°C) and were given approximately 18 hours to adhere to the Mylar^®^ surface before radiation exposure.

Sterilization of the individual cup components was accomplished by placing all components in a 500 mL bath of 100% ethanol for 30 minutes, agitating with a stir bar at 300 rpm. The components were rinsed in sterile 1X Phosphate Buffered Saline (PBS) and allowed to dry in a standard biological safety cabinet. The Mylar^®^ sheets were submerged in a bath of 100% ethanol for one minute, then rinsed with 1X PBS and allowed to dry.

Cup assembly consisted of a Mylar^®^ sheet (Fig 1A) being stretched over the outer cup component (Fig 1C). The inner cup component (Fig 1B) was then positioned over the opening of the outer cup component and was firmly pressed together until a snap was heard. The cups were then closed with a cap that allowed for ventilation (Fig 1D).

### X-ray irradiation and dose calculation

#### Irradiation setup and procedure

**Fig 2:**
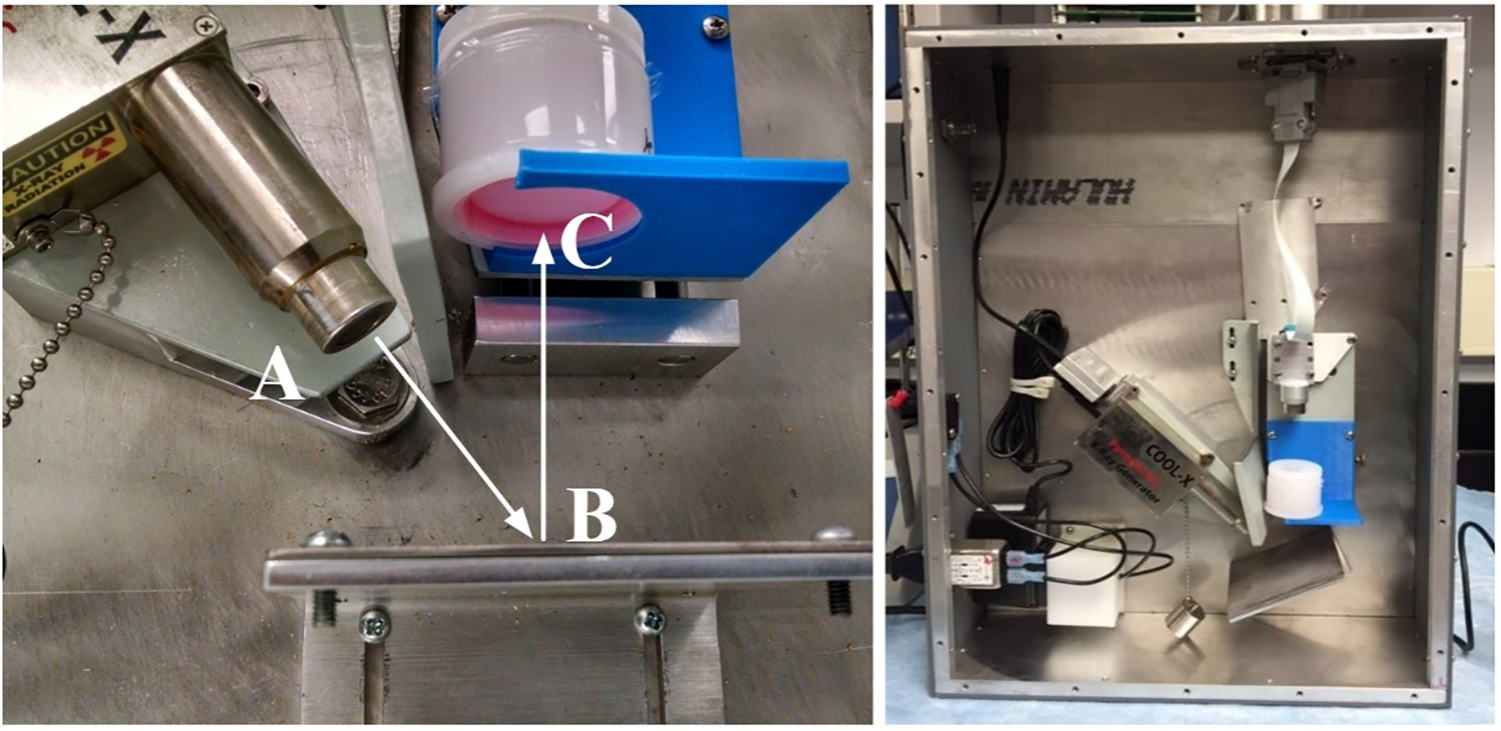
X-ray irradiator setup. The radiation source (A) targeted the cell culture by fluorescing off the metal plate (B) before irradiating the culture through the Mylar^®^ from the base of the cup (C).

In order to facilitate the irradiation studies, we have developed a novel x-ray fluorescence (XRF) irradiation setup with an x-ray generator producing characteristic iron (Fe) and Bremsstrahlung x-rays [28] and an x-ray detection system based on single photon counting, shown on Fig 2.

Cell cultures in the Mylar^®^ cups were exposed to the Kα and Kβ emissions of the target metal by being placed in a holder directly over an x-ray fluorescence metallic plate. The x-ray generator with a 120-degree emission cone [29] shined x-rays onto the metal plate directly, out of range of the cell culture, which was further shielded in order to assure that the cell culture was only exposed to the dichromatic spectrum consisting primarily of the Kα and Kβ fluorescent emission lines of the metal. By exposing the cell culture to these characteristic x-rays over a period of time and applying single photon detection based dose rate calibration procedure, an accurate radiation dose can be delivered to the cells.

**Fig 3:**
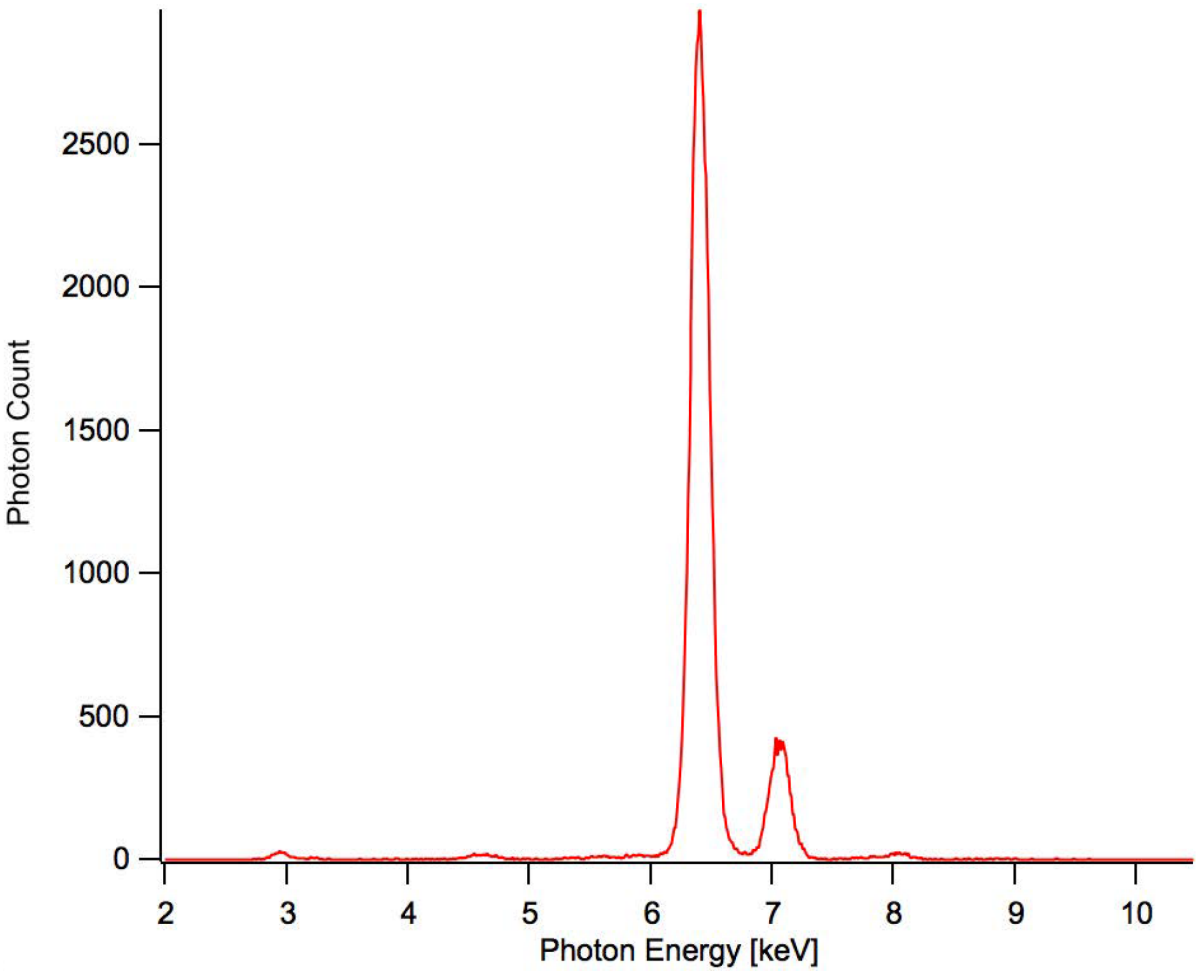
Iron (Fe) XRF irradiation spectrum. Displaying photon count vs. photon energy [keV].

The NIH 3T3 fibroblast cell samples of the current study were irradiated using an iron (Fe) fluorescent plate emitting x-rays of 6.40 keV and 7.06 keV respectively (Fig 3). The relative intensities of the Fe and emissions were approximately 5 to 1. Each sample was irradiated for one hour resulting in a total dose of 551 μGy ± 119 μGy. The uncertainty was largely due to the variation of the intensity of the x-ray generator used and can be significantly improved in further studies [30].

**Fig 4:**
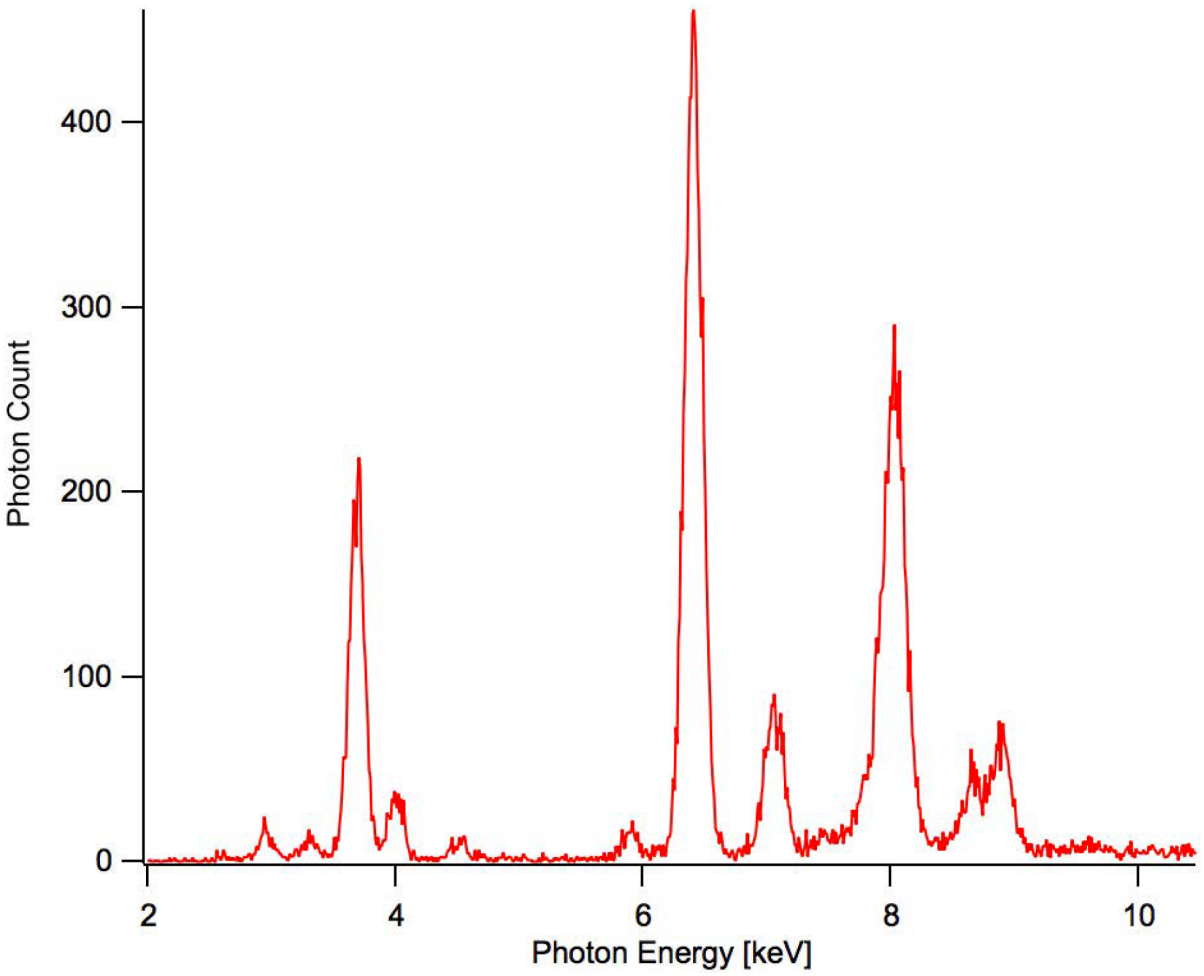
Calibration spectrum. Photon counts vs. photon energy [keV] of the SDD detector exposed to characteristic XRF emissions from a multivitamin target material for 900 seconds.

Dose rates were determined prior to irradiation by positioning a silicon drift detector (SDD) capable of detecting individual x-ray photon hits at the cell sample location. X-ray spectra were measured for several one-hour trial periods and the dose rates were determined by calculating the total energy deposited into the detector by adding up the energy of the single hits. The difference in geometries between the detector and the cell samples along with the transmission and absorption rates were taken into account for the dose-rate calculations. For the calibration of the x-ray photon energies, a calibration plate was coated with multivitamin powder to create multiple well-defined peaks of various energies (Fig 4). The nominal peak energies were obtained from the NIST X-Ray Transition Energies Database [31].

The fibroblast culture was placed above the fluorescent plate so that the x-rays were sent through the base of the Mylar^®^ cup (Fig 2) with very small attenuation. This irradiation procedure was implemented and performed for a series of individual samples to complete the cell proliferation assay, bicinchoninic acid assay, and cell cycle analysis using flow cytometry.

#### Dose deposition and calculation

The absorbed dose is the expectation value of the energy imparted to matter per unit mass at a point [32]. Due to the low-energy of the incident photons within this experiment, the dominating initial interactions taking place within the cell-cultures are photoelectric in origin. The ejected photoelectron will further deposit energy into the surrounding material by colliding with and ionizing atoms and molecules [32].

Calculating dose accurately requires calculating the collisional amount of kinetic energy released in mass, or kerma [32]. This was done using the calculated energy deposition into the SDD detector over several test periods that were identical to the experimental bio-irradiation runs. The data showed that the two characteristic x-ray peaks of the energies from the iron surface (6.40 keV and 7.05 keV, respectively) were the only peaks being detected.

An average of about 86,000 photons and 11,500 photons (6.40 keV and 7.05 keV respectively) were counted by the detector during the trial periods and as a result of the high counting statistics the pulsing nature of the x-ray generator has been determined to contribute nearly all of the uncertainty of the dose calculations, with a standard deviation of σ = ± 28%.

Since the collection area of the SDD detector is less than the flux area of the cell-culture dish, the flux was scaled up by a factor of roughly 45 to account for this difference with uniform field of fluoresced photons assumed across the dish.

The equation of the collision kerma containing multiple characteristic x-rays is

$$K_{c}=\sum_{i=1}^{n}E_{i}\frac{dN}{dA}\left(\frac{A_{cup}}{A_{det}}\right){\left(\frac{\mu_{en}}{\rho }\right)}_{E_{i},Z}$$ (1)

Interpolating data provided by the NIST database, the mass-energy coefficients for the photons (6.40 keV and 7.05 keV) in ICRU-44 soft tissue equivalence were calculated to be 21.2 cm^2^/g and 16.3 cm^2^/g respectively.

It is critical to note whether the charged particle that has obtained kinetic energy from the incident photon stays in the material or is ejected altogether. In the case of this experiment, as the photon-matter interaction is dominated by the photoelectric effect, the average range that an electron imparted with the maximum available kinetic energy can travel within the medium has been determined with the following equation:

$$<t>={\left(\frac{R_{CSDA}}{\rho }\right)}_{Z}^{E}$$ (2)

Here < *t* > is the average range in *cm*, *R*_*CSDA*_ is the continuous slowing down approximation within a material (*Z*) for an electron of a certain energy (*E*) in units of *g*/*cm*^2^, and ρ is the density of the material. For 10 keV electrons in ICRU-44 soft tissue, the values given by NIST concerning these specific parameters yield a length of only 2.5 microns. For electrons of lower energy than this, the range shortens exponentially. This allows the assumption that all the photons incident within the material will have the entirety of their energy imparted to the material as well (the monolayer of cells is about 2–3 μm in height). Thus, we can confidently calculate the integral dose as being equal to the calculated collisional kerma, *K*_*C*_ = *D*.

Considering the above the average integral dose delivered to each of the irradiated bio-sample volumes was calculated to be 551 μGy ±119 μGy. This yields an effective dose during the full exposure roughly equal to that of 3 to 7 diagnostic chest x-rays [33].

### Cell culture analysis after irradiation

Analysis of cell cultures following irradiation were done in 24 hour intervals where day 0 refers to a test immediately following irradiation exposure and day 1 refers to the first 24 hours after the irradiation exposure has been performed.

Cell proliferation assays were performed over a period of four days. Cell proliferation was measured using CellTiter96^®^ Aqueous One Solution (Promega, Madison, WI), a colorimetric assay that quantifies the formazan product released by cells in culture. Formazan is directly proportional to the amount of cells present in a culture, where a lighter colored solution is indicative of less cells compared to a darker colored solution. By studying the change in cell proliferation over a period of four days, the significance in cell concentration between the control and non-irradiated cultures can be observed. The testing solution was placed in a 96 well plate in triplicates and measured for absorbance at 490 nm using a Synergy H1 Biotek plate reader. Each group (e.g., Irradiated and Control at each time point) included data from 4 separate cell culture samples (N = 4).

Total protein analysis on four irradiated and four control fibroblast samples were performed with the Pierce^™^ BCA Protein Assay Kit (ThermoFisher Scientific, Waltham, MA) (N = 4). Similar to the proliferation assay, the BCA Assay is a colorimetric assay that determines the density of protein produced by the cells based on the measured absorbance of the solution. Lysate collection for the BCA assay was performed at 24 hour intervals beginning the day following the radiation exposure for a period of four days. The cells were trypsinized and placed into 15 mL centrifuge tubes. After removing the supernatants, the cells were resuspended in 150 μL room temperature Mammalian Protein Extraction Reagent (MPER; ThermoFisher Scientific) in order to collect the lysates. The lysates were mixed with a working reagent consisting of Pierce BCA Protein Assay Reagent A/Reagent B, which measures the reduction of Cu^+2^ to Cu^+1^ in proteins. The resultant solution, which would turn various shades of purple, was then placed in a 96 well plate in triplicates before being tested for absorbance at 562 nm in a Synergy H1 Biotek plate reader.

For Flow Cytometry analysis, samples at day 0 refer to a set of controls fixed following plating and cell attachment to the Mylar^®^ surface, where day 1 refers to the first time point 24 hours after irradiation exposure. A total of 18 samples were required to perform flow cytometry, which is an assay that determines the percentage of cells in G_0_/G_1_, S, and G_2_/M phases of the cell cycle. Flow cytometry could be used to determine which stage of the cell cycle the irradiated and control cells spend most of their time. Six samples were irradiated, and six served as the corresponding controls for 3 days. The remaining five samples were controls for Day 0. 3T3 fibroblasts were plated in each cup at approximately 5,500 cells/cm^2^. On each respective day, cells were trypsinized, centrifuged, fixed in ice cold 70% ethanol, and stored at -20°C in order to suspend the cell cycle. Fixed cells were pelleted and rinsed with PBS several times, then resuspended in 200 μL of Guava Cell Cycle reagent (EMD Millipore, Darmstadt, Germany). A Guava easyCyte Flow Cytometer was used to gather data about the cell cycle.

DAPI (ThermoFisher Scientific) and phalloidin (ThermoFisher Scientific) were the stains used to stain the nucleus (blue) and the actin cytoskeleton (green) of each fibroblast cell. These stains help demonstrate the internal structure of the cells to give a qualitative representation of any visible changes based on the irradiation. A primary antibody of mouse anti-collagen type I (Hybridoma Bank, University of Iowa, Iowa City, IA) was used to stain for collagen protein (red) with a secondary of goat anti-mouse Alexa Fluor 647 (ThermoFisher Scientific). Cells were imaged using a Thermo Fisher EVOS^®^ FL Imaging System at an objective of 40x.

All statistical analyses were calculated using a Student’s T-test, using an α < 0.05 for statistical significance.

## Results and discussion

### Cell proliferation

**Fig 5:**
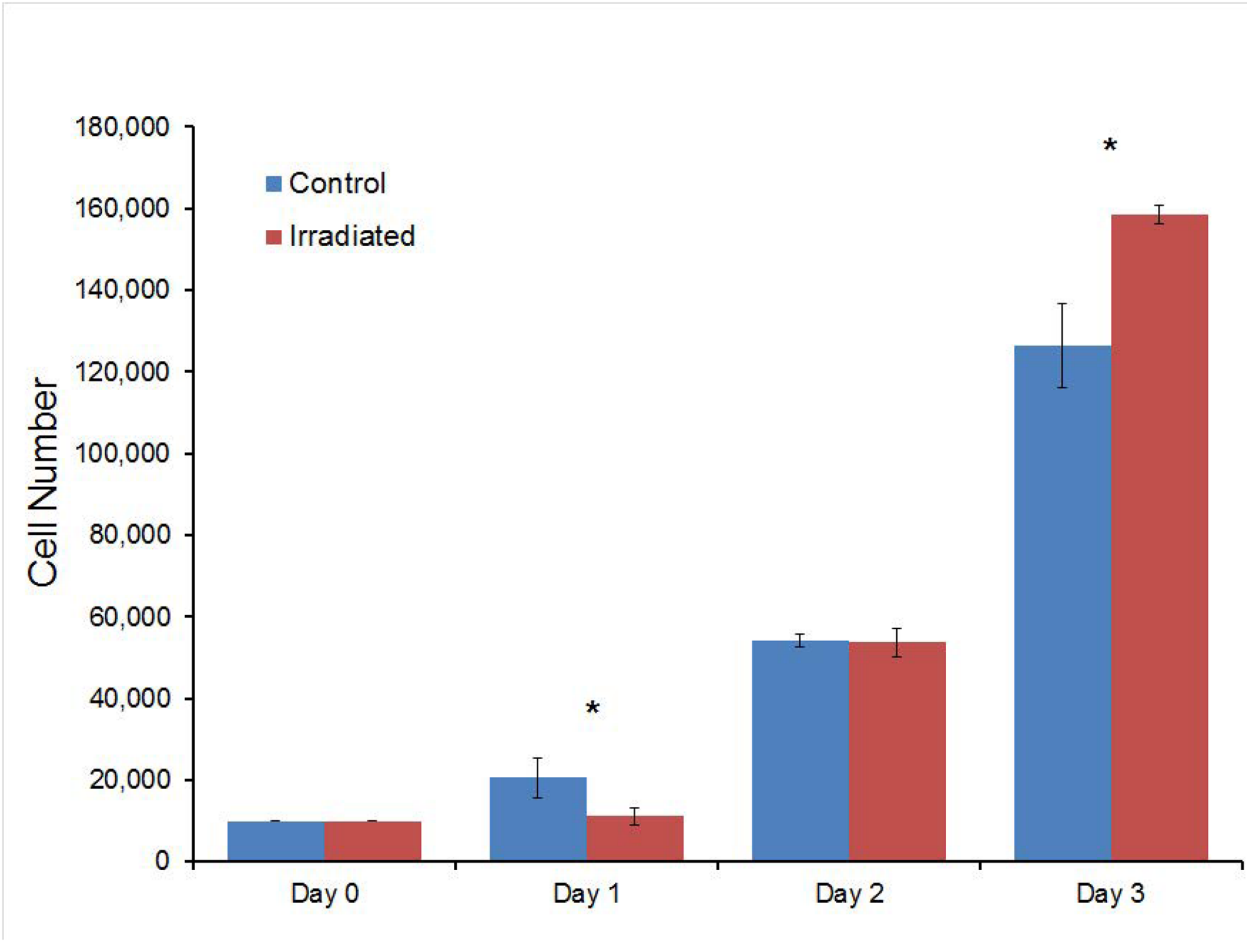
Proliferation assay. Total cell number increased over time in culture for both the irradiated and control groups. However, while cells in the irradiated group had initially lower cell numbers one day after treatment (* = p<0.05), they proliferated much more quickly in the days following.

After irradiation, the proliferation assay showed increasing cell numbers for a period of up to 3 days (Fig 5). Cultures in both the irradiated and control groups were visibly 95% confluent by Day 5. At Day 1, the irradiated samples displayed slower proliferation and appeared to pause at 11,000 cells, while the controls nearly doubled in concentration. By Day 3, the samples showed a statistically significant difference (p = 1.45e-5) in proliferation rate as they surpassed the controls by approximately 30,000 cells.

The initial difference in cell number (Fig 5) is likely due to the radiation inhibiting proliferation for a period of time, possibly as a protective measure to minimize DNA damage [24, 34]. Applied stresses to cell cultures can create a lag phase before proliferation such as in the case of trypsinization or thawing which could explain the pause in proliferation of the irradiated samples. The increased proliferation rate of the irradiated cells following the pause in proliferation could be due to the cells speeding up the cell cycle. The cells that proliferate the fastest can thus produce protein at a higher rate. However, the ultra-low doses of x-ray used in this experiment are unlikely to cause significant cell apoptosis or double strand DNA damage [35, 36]. In addition, since both control and irradiated cultures do not qualitatively reach near confluency until much later time points (Day 5), it seems unlikely that the increased proliferation rate seen in the irradiated group at days 2 and 3 is due to “uncrowding” or confluency of the cultures in this experiment.

### Changes in total protein content

**Fig 6:**
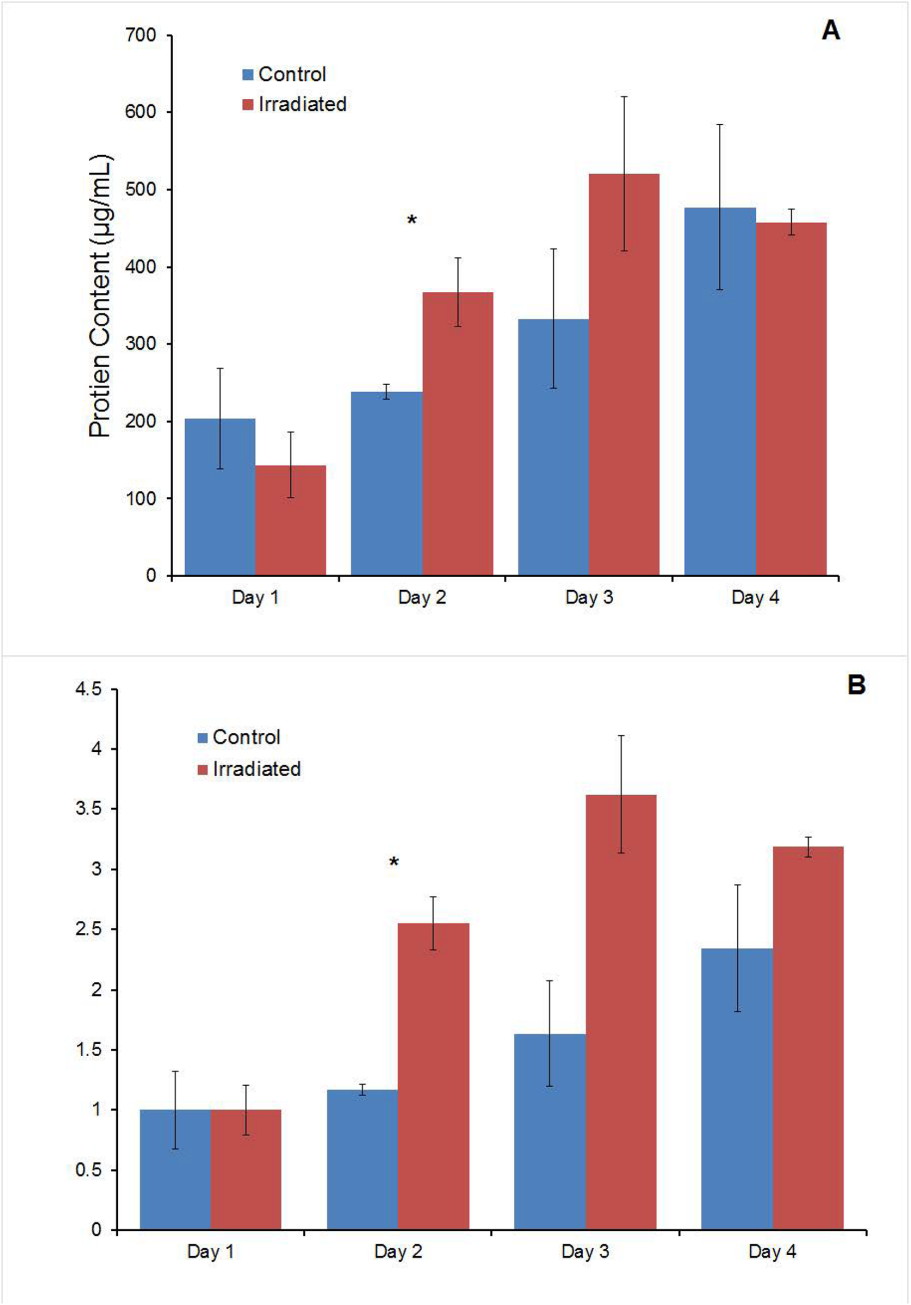
Total protein content. (A) BCA Analysis over 4 days after irradiation (p<0.05). (B) The normalized data shows the change in protein levels increased more in the irradiated group than in the control, following a similar trend to that of the Proliferation Data.

The BCA analysis after radiation exposure over four days showed increases in total protein content present in the irradiated cultures (Fig 6). Irradiated cells on Day 1 expressed a lower amount of protein in comparison to its control counterpart. By Day 2, the irradiated cells surpassed the control by about 130 μg/mL and continued to surpass the control by Day 3; however, no statistically significant difference was observed.

The increase in protein level at Days 2 to 4 is consistent with prior *in vitro* and *in vivo* studies that showed that ultra-low dose radiation could increase cell proliferation, since an increase in cell proliferation would likewise indicate an increase in protein production as well [33, 37]. Furthermore, the data suggests that irradiation at this dose does not damage the cells’ ability to produce proteins.

### Cell cycle analysis

**Fig 7:**
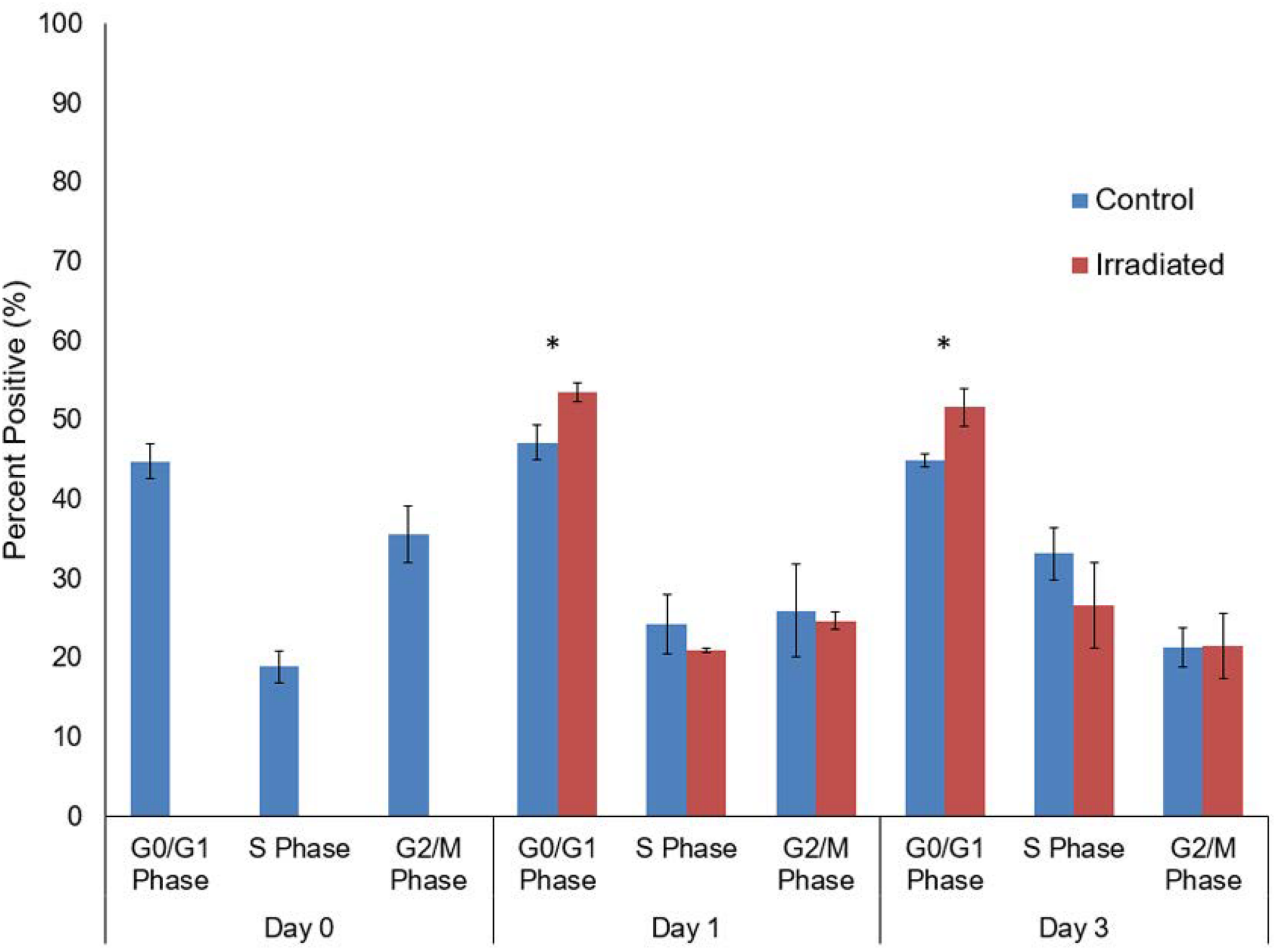
Cell cycle analysis using flow cytometry. Cells were measured to determine the percent positive in which they resided in the G_0_/G_1_ phase, S phase, and G_2_/M phase of the cell cycle (* = p<0.05).

Cell cycle analysis showed that in comparison to their controlled counterparts, the irradiated cells had a higher percentage in the G_0_/G_1_ phase (Fig 7). However, the irradiated cells had a smaller percentage in the S and G_2_/M phase in comparison to their controls, implying that less time was spent in those stages of the cell cycle.

The results of the cell cycle assays (Fig 7) are consistent with prior studies which have shown that low dose (0.1 Gy) x-rays can temporarily arrest the cell cycle of human mesenchymal stem cells in G_0_/G_1_ phase [38]. Since DNA becomes most vulnerable during the G_2_/M phases of cell cycle where the nuclear membrane deteriorates, it is thought that by retaining the nucleus around the DNA, the cell refrains from exposing its DNA to the potentially harmful environment created by the x-rays.

The low dose x-ray radiation could have some effect on the genes that regulate the mitotic stages of the cell cycle, causing them to express senescence, which occurs when the cells remain in the G_0_ phase to keep their DNA intact and protected [39]. After the radiation period and once any DNA damage has been repaired, the cells could begin to proliferate again. The data recorded in Figs 5 and 7 emphasize this assumption, given that a significantly larger percentage of irradiated cells were found to spend their time in the G_0_/G_1_ phases during day 1 and 3 compared to the controls. For day 3, however, we would have expected to see a significant increase in the percentage of cells in the G_2_/M phase in Fig 7, which would match the high proliferation rates in Fig 5. However, by day 3 for Fig 7, the irradiated cells continued to spend a statistically significant amount of their time in the G_0_/G_1_ phase. Though there was an increase in the percentage of the irradiated cells in the S phase between days 1 and 3, this percentage did not outnumber the control, suggesting that irradiation might potentially affect the cell cycle negatively by creating a form of unhealthy proliferation that does not go through these checkpoints (Fig 7). More studies should be done to fully characterize the effects of irradiation on cell proliferation to see if this proliferation is healthy. Assays such as PCR to examine specific gene expression would be ideal for future tests.

### Immunofluorescence imaging

**Fig 8:**
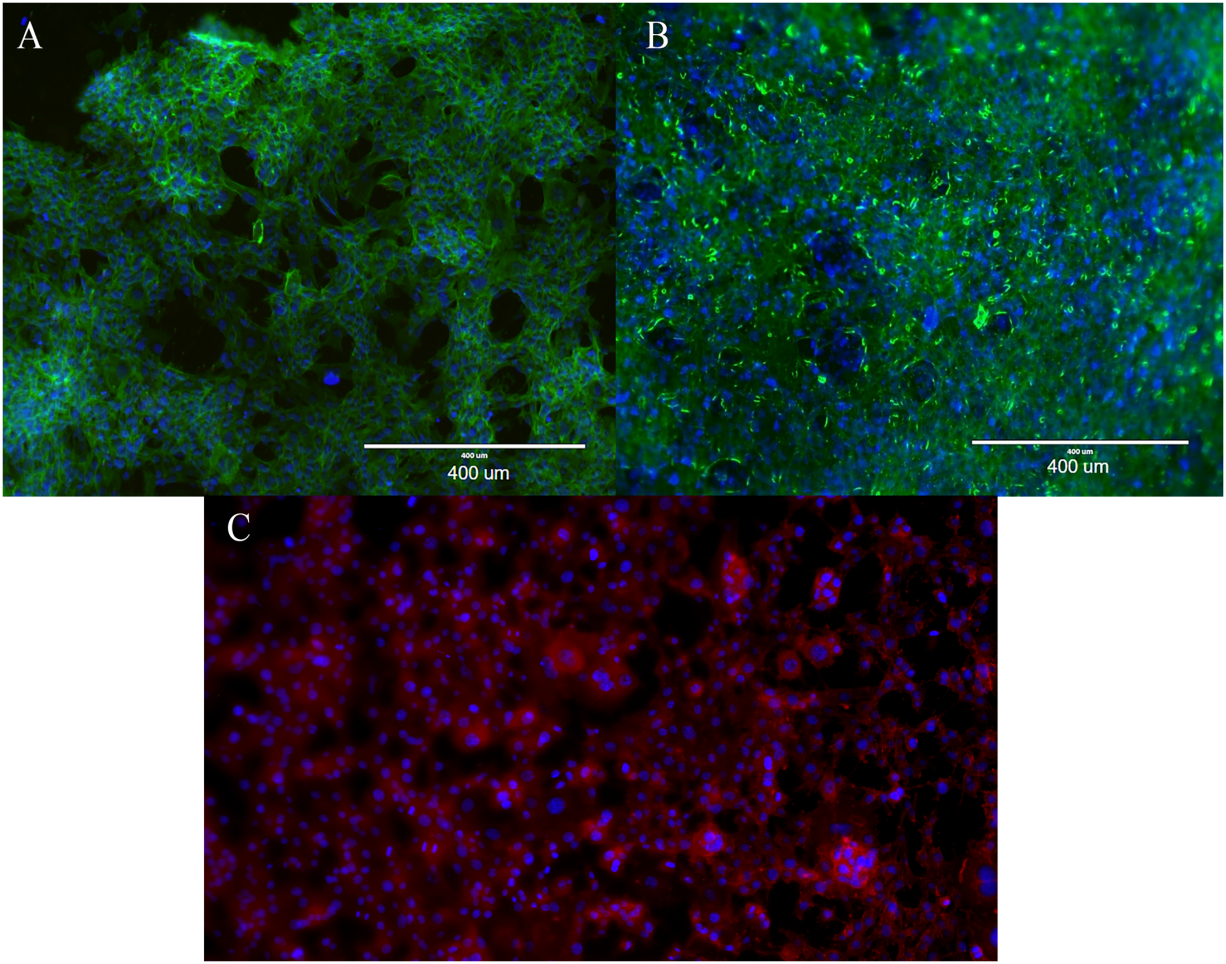
Cell staining. After irradiation, control samples (A) and irradiated samples (B) were stained for collagen (red), nuclei (blue), and cells (green) at 10X magnification on Day One after irradiation. Irradiated cells (C) stained to show collagen production.

Immunofluorescent images of the cells (Fig 8) confirmed the findings of the cell proliferation assay; more cells were observed at Days 2–4 in the irradiated samples than control samples. In addition, cells in the irradiated samples showed staining for type I collagen that was similar to the control sample. This suggests that the irradiated cells still maintained their normal cell functions after radiation exposure since there were no visible differences between the cell morphology and collagen staining of control and irradiated fibroblast cultures.

## Conclusions

The new tunable monochromatic low-dose x-ray cell irradiation system presented here reduces the complexity and cost of cell radiation experiments, which opens new avenues of study. For instance, this system can allow researchers to investigate the effect of x-ray wavelength on cells. The results of low dose, well-characterized irradiation studies could help develop new ways of utilizing low dose radiation in clinical settings, as well as fully determine specific dose and dose rate thresholds for certain outcomes. Further studies will help determine the full breadth of benefit and harm that monochromatic, low-dose, soft x-rays can have.

We have employed a novel, well-characterized x-ray fluorescence setup to irradiate 3T3 fibroblast cells with a low-dose of soft x-ray radiation. The total dose of radiation consisted of 6.5 keV and 7.05 keV monochromatic photons fully depositing their energy within the volume of the cell culture and the surrounding media. Although x-rays have a much lower LET in comparison to charged particle sources, the low energy x-rays used in this study have a higher LET than many of those used for diagnostic imaging and therapeutic devices. Thus, the amount of ionizations and potentially damaging events within the cell are higher than those typically used in many medical applications. However, because clinical x-ray sources produce a broad spectrum of x-rays, the energies used here are within the spectrum of the x-ray energies that are emitted from some clinical sources (i.e. Molybdenum sources used for mammography [40]). Most radiation sources used in biological experiments are not fully characterized. In most studies, the only properties reported about the source are the method of generating the radiation (linear accelerator, x-ray tube, elemental decay, etc) and the total dose applied. In some studies, the dose rate is also reported. Our system has opened up the possibility of more fully characterizing the applied radiation, and in turn being able to determine if different wavelengths and energies, or methods of generating radiation have significantly different effects on their biological targets.

The dose used in this study is similar to the x-ray dose received by tissues during standard clinical x-ray imaging at higher x-ray energies [41, 42] and astronauts in low-earth orbit [7]. The organ dose limit recommended by the National Council on Radiation Protection and Measurements for air flight personnel and low-earth orbit astronauts is 250 mSv- 1.5 Sv over 30 days. If this dose is reached through continuous exposure, this translates to an exposure rate of 0.35–2 mSv/hr. The dose rate from the irradiation system described here was set to 0.55 mGy/hr to be within this range but it should be noted that the dose rate can easily be varied by changing the power to the x-ray source (Fig 2). By allowing for independent control of parameters, this novel x-ray irradiation system useful for further studies studying the effect of total dose, dose rate, and x-ray wavelength on cell cultures [43].

The increased proliferation rate and protein concentration for the irradiated 3T3 fibroblast cells at this dose rate suggests that very low dose soft x-ray radiation might cause an initial pause in cell proliferation followed by a significant increase cell proliferation, which is consistent with prior studies using similar x-ray total doses on other cell types [25]. While the exact mechanism for this phenomenon is currently unknown, it can now be further studied systematically as a function of the properties and characteristics of x-rays using the new system described here [26]. These tests further prompt the investigation of more specific responses that cause proliferation change by using various doses and various spectral distributions of radiation on several cell types.

## Supporting information

S1 Excel Excel version of the data for Figs 5–7. (RAR)
